# Conjugated Polymer Controlled Morphology and Charge Transport of Small-Molecule Organic Semiconductors

**DOI:** 10.1038/s41598-020-61282-x

**Published:** 2020-03-09

**Authors:** Zhengran He, Ziyang Zhang, Sheng Bi, Jihua Chen, Dawen Li

**Affiliations:** 1 Department of Electrical and Computer Engineering, The University of Alabama, Tuscaloosa, AL 35487 USA; 2 Department of Electrical Engineering, Columbia University New York City, New York City, NY 10027 USA; 3 Key Laboratory for Precision and Non-traditional Machining Technology of the Ministry of Education, Dalian University of Technology, Dalian, Liaoning 116024 China; 4 Center for Nanophase Materials Sciences, Oak Ridge National Laboratory, Oak Ridge, TN 37831 USA

**Keywords:** Electronic devices, Electronic and spintronic devices

## Abstract

In this study, we report an effective approach to tune the crystallization, microstructure and charge transport of solution-processed organic semiconductors by blending with a conjugated polymer additive poly(3-hexylthiophene) (P3HT). When 6,13-bis(triisopropylsilylethynyl) pentacene (TIPS pentacene) was used as a model semiconductor material to mix with different amount of P3HT, their intermolecular interactions led to distinctive TIPS pentacene film morphologies, including randomly-oriented crystal ribbons, elongated needles with enhanced long-range order, and grass-like curved microwires with interlinkages. Each type of morphology was found to further correlate to considerably different charge transport and device performance. As compared to pristine TIPS pentacene devices, bottom-gate, top-contact OTFTs with 2% in weight P3HT additive showed a 2-fold and 5-fold improvement of average field-effect mobility and performance consistency (defined as the ratio of average mobility to the standard deviation), respectively. The improvement in transistor electrical performance can be attributed to the combined effect of enhanced crystal orientation and uniformity, as well as increased areal coverage. This work can be applied beyond the particular example demonstrated in this study and to tune the charge transport of other small-molecule organic semiconductors in general.

## Introduction

In recent years, the research of high-performance, solution-processed semiconductor based organic thin film transistors (OTFTs) has attracted great attention^1–6^. In particular, substantial progress has been reported in the study of film morphology and charge transport of various small-molecule organic semiconductors, such as *N*,*N*’-1*H*,1*H*-perfluorobutyl dicyanoperylenecarboxydiimide (PDIF-CN_2_)^7,8^, 6,13-bis (triisopropylsilylethynyl) pentacene (TIPS pentacene)^9,10^, and 5,11-bis(triethylgermylethynyl) anthradithiophene (diF-TEG-ADT)^11,12^, which enables the field-effect mobility to reach or even surpass that of amorphous silicon^13^. Notwithstanding these advances, the fabrication of high-mobility organic semiconductor crystals typically requires the method of slow crystallization in solution, which inevitably results in randomly-oriented crystals^14,15^, and further leads to anisotropic charge transport and significant device-to-device performance variations of OTFTs^16^.

Various external alignment techniques, such as air flow navigation^17^, temperature gradient^18^ and substrate tilting^19^, have been demonstrated to control the growth and align crystal orientation of small-molecule organic semiconductors. Besides, the approach of blending with polymer additives, which does not require the complex setup of external alignment techniques, has been reported as a simple and effective pathway to modulate the semiconductor crystallization, tune thin film morphology and enhance charge transport^20^. For example, isotactic poly(methyl methacrylate) (*i*-PMMA) was blended with TIPS pentacene, and in conjunction with a vertical flow approach, the *i*-PMMA additive improved the semiconductor crystallization and OTFT performance^21^. A series of polyacrylate polymers with different length of hydrophobic side group were blended with TIPS pentacene and led to a lateral or vertical phase segregation, which was found to depend on the polymer side group length^22^. Also, poly(α-methyl styrene) (PαMS) was mixed with TIPS pentacene to tune crystallization and charge transport of the semiconductor^23^, resulting in a vertical phase segregation and improved mobilities of OTFTs^24,25^. However, previous works which studied polymer/semiconductor blends were overwhelmingly dedicated to amorphous polymers, whereas little information has been reported regarding the systematic effect of increasing conjugated polymer additives on tuning the thin film morphology and charge transport of the small-molecular semiconductors.

In this work, we report the effect of increasing content of conjugated polymer additive poly(3-hexylthiophene) (P3HT) to tune the crystallization and morphology of small-molecular organic semiconductors. With TIPS pentacene selected as a model semiconductor material to blend with P3HT, we demonstrate that the intermolecular interactions between these two components led to distinguishing TIPS pentacene film morphology. Different morphology patterns of TIPS pentacene from different P3HT loading ratios were newly reported, including randomly-oriented crystal ribbons, well-aligned needles with enhanced long-range order, and grass-like curved microwires with interlinkage, which were further correlated to distinctive charge transport and device performance of the TIPS pentacene based transistors. Bottom-gate, top-contact OTFTs with the addition of P3HT polymer at 2% weight ratio showed a 2-fold and 5-fold enhancement of average mobility and performance consistency factor (defined by the ratio of average mobility to standard deviation of mobility), as compared to pristine TIPS pentacene devices. This remarkable enhancement of OTFT device performance can be attributed to the improved crystal orientation and areal coverage as a result of the addition of P3HT conjugated polymer additive. This work can be facilely utilized to optimize the electrical performance of other small-molecule organic semiconductors.

## Experiment

Small-molecule semiconductor TIPS pentacene was purchased from Sigma Aldrich and was used without further purification. Conjugated polymer additive P3HT was purchased from Rieke Metals (BASF Sepiolid P200, M_n_ of ~20000 g/mol by Gel Permeation Chromatography). Solvent toluene was purchased from VWR and was directly used as purchased.

Both TIPS pentacene and P3HT were dissolved in toluene at a solute concentration of 5 mg/ml, and P3HT was mixed with TIPS pentacene at the following weight ratios of P3HT: 2%, 10%, 25% and 50%, respectively. Then the TIPS pentacene/P3HT mixture was drop casted onto a heavily doped *n*-type silicon substrate with a 250 nm thickness of silicon dioxide. The TIPS pentacene crystals were grown in a solvent-rich environment by placing the substrate inside a covered petri dish. The substrate was tilted with a very small angle (<5 degrees) in order to facilitate the formation of oriented TIPS pentacene crystals. A typical slow crystallization process in solution takes 0.5~1 hour.

Transistor fabrication was based on a bottom-gate, top-contact configuration. After the formation of TIPS pentacene crystals as the semiconducting channel, 50 nm of gold (Au) was deposited through a shadow mask as source and drain contact electrodes by using a thermal evaporator (Angstrom Engineering) with a deposition rate of 1 Å/s at a pressure of 10^−7^ Torr. The channel length was fixed at 2000 μm, whereas the channel width varied as 25 μm, 50 μm, 75 μm and 100 μm, respectively.

Thin film morphology of TIPS pentacene crystals was characterized by using a polarized optical microscope. Electrical characterization of TIPS pentacene based OTFTs, including both transistor output (*I*_*DS*_ − *V*_*DS*_) and transfer (*I*_*DS*_ − *V*_*GS*_) characteristics, was measured using a Keithley 4200 semiconductor parameter analyzer. All measurements were conducted in an ambient condition at room temperature. OTFTs were tested five times to ensure the consistency of measurement.

## Results and Discussion

**Figure 1:**
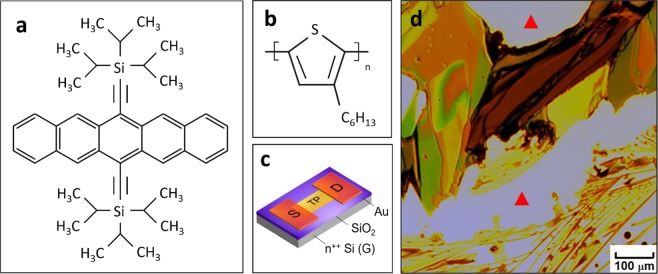
Molecular structure of (**a**) small-molecule organic semiconductor 6,13-bis (triisopropylsilylethynyl) pentacene (TIPS pentacene); and (**b**) conjugated polymer additive poly(3-hexylthiophene) (P3HT). (**c**) Device configuration of the bottom-gate, top-contact TIPS pentacene based transistors. “S” and “D” indicates the “source” and “drain” gold contact electrodes. “TP” indicates “TIPS pentacene”. (**d**) A microscopic optical image of pristine TIPS pentacene film. The bare substrate not covered by TIPS pentacene is indicated by the red triangles.

6,13-bis(triisopropylsilylethynyl) pentacene (TIPS pentacene) was chosen as a benchmark material to blend with P3HT in this work. TIPS pentacene is a *p*-type organic small-molecule semiconductor that has been extensively studied for its high charge transport, excellent air stability and solution processability^26,27^, and finds application in various organic electronics devices, including transistors and photodetectors^28–30^. As shown in the molecular structure of Fig. 1(a), TIPS pentacene has two bulky side groups, which disrupt the pattern of herringbone packing and enhance its solubility^31,32^. In addition, its enhanced π-π stacking improves the charge transport in TIPS pentacene^33–35^. The molecular structure of the polymer additive P3HT is shown in Fig. 1(b).

**Figure 2:**
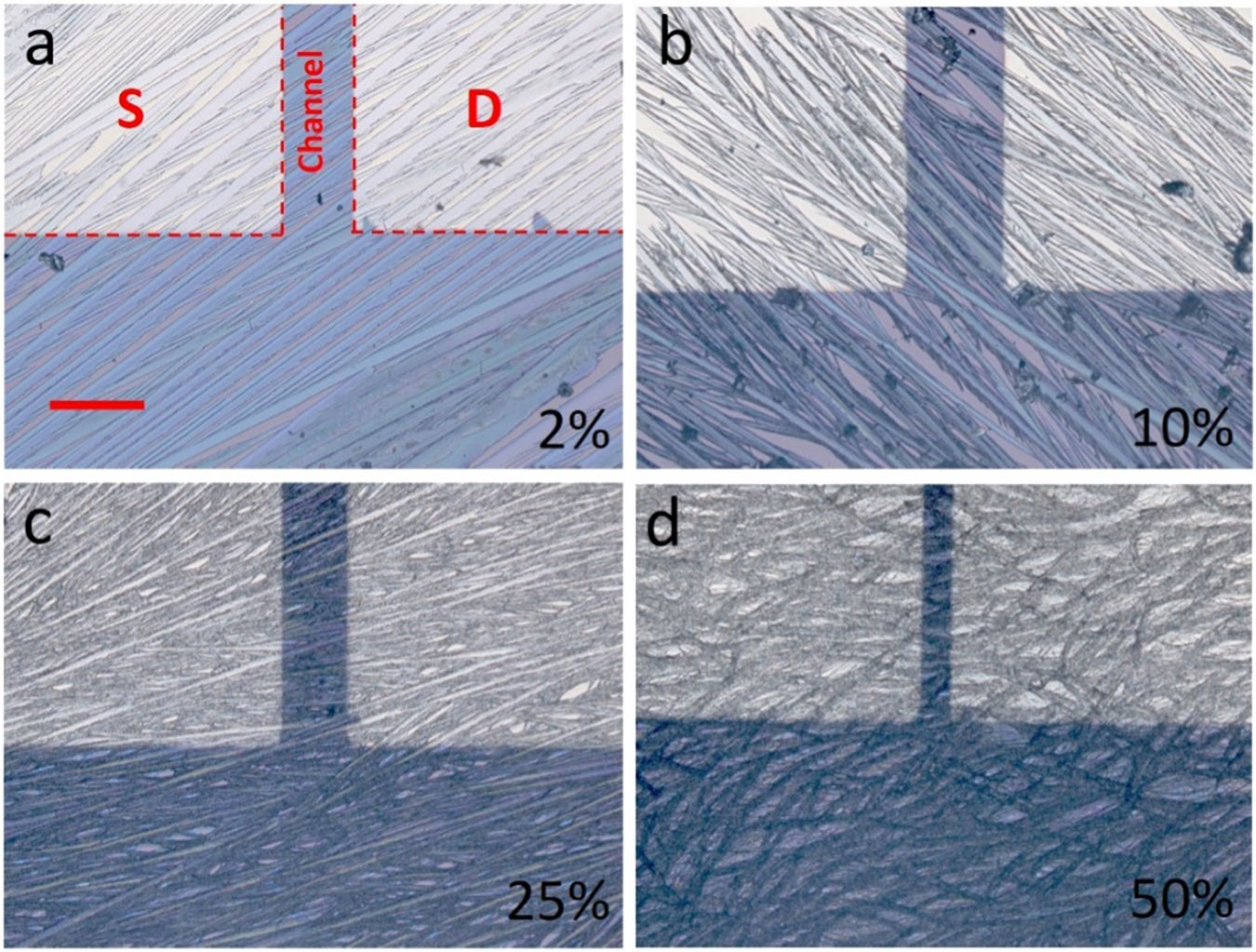
Polarized optical microscopic images of TIPS pentacene/P3HT blend films with different P3HT weight ratios: (**a**) at 2%, elongated TIPS pentacene crystals were formed along a uniform direction with enhanced long-range order; (**b**) at 10%, crystal gaps were enlarged with more misorientation; (**c**) at 25%, TIPS pentacene film exhibited a combination of both crystal needles and curved microwires; and (**d**) at 50%, grass-like, curved microwires dominated the film morphology. “S” and “D” in (**a**) indicates the “source” and “drain” gold contact electrodes. All optical microscopic images share the same scale bar of 100 μm as in (**a**).

In order to tune the TIPS pentacene morphology, conjugated polymer additive P3HT was blended with TIPS pentacene at different weigh ratios, including 2%, 10%, 25% and 50%, respectively. The pristine TIPS pentacene formed randomly oriented crystals when grown via the method of drop casting, which resulted in anisotropic charge transport and significant device-to-device performance inconsistency of TIPS pentacene based OTFTs^17^. The microscopic optical image of pristine TIPS pentacene film is shown in Fig. 1(d). When a small amount of P3HT (i.e. at 2% weight ratio) was blended with TIPS pentacene, it led to the formation of needle-shaped crystals with enhanced crystal orientation and areal coverage across the entire substrate, as shown in the optical images of Fig. 2(a). This type of morphology with improved long-range order is beneficial for charge transport and device performance of TIPS pentacene based OTFTs. As the P3HT ratio increased to 10%, the TIPS pentacene needles exhibited more intermixing and enlarged crystal gaps, as shown in Fig. 2(b). In addition, curved crystal microwires started to appear at a P3HT weight ratio of 25%, leading to a mixture of both elongated crystal needles and microwires (Fig. 2(c)). Finally, when the weight ratio of P3HT increased to 50%, the TIPS pentacene film dominantly exhibited curved, grass-like microwires with interlinkage, as shown in Fig. 2(d). Although substrate tilting (<5 degrees in this work) was reported to facilitate the formation of oriented TIPS pentacene crystals^19^, such alignment was not observed in the pure TIPS pentacene film without blending P3HT. Therefore, we attribute the improved crystal alignment mainly to the addition of the P3HT conjugated polymer.

**Figure 3:**
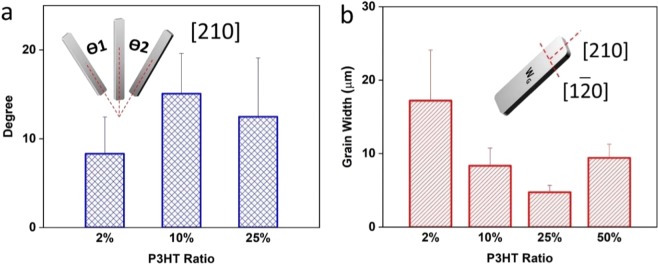
Plot of (**a**) the misorientation angle (θ), and (**b**) grain width of TIPS pentacene film at different weight ratios of P3HT. As shown in the inset of (**a**): a baseline crystal was first selected, and the misorientation angle (θ_1_ or θ_2_) is defined as the angle between the long axis [210] of the baseline crystal and that of another crystal. The grain width, or *W*_*G*_, as denoted in the inset of (**b**), is defined as the domain width along the short axis [1$\overline{2}$0] of a crystal. The average and standard deviation of both misorientation angle and grain width are based on the measurement of 10 selected, representative crystals.

As can be seen from the optical images of Fig. 2, the addition of P3HT conjugated polymer additive effectively tuned both crystal orientation and grain width of TIPS pentacene film. Therefore, we quantitively measured crystal misorientation angle and grain width as a function of different P3HT loading ratios. As the inset of Fig. 3(a) shows, a TIPS pentacene crystal was first selected as a baseline crystal, and the crystal misorientation angle (θ) of a particular TIPS pentacene crystal was defined as the angle between the long axis [210] of this crystal and that of the baseline crystal. Apparently, a smaller misorientation angle indicates improved crystal orientation. While the pristine TIPS pentacene film has a large misorientation angle of 41.4° ± 27.1°^17^, indicating the crystals were randomly oriented, the addition of P3HT at a weight ratio of 2%, 10% and 25% led to a decreased misorientation angle of 8.3° ± 4.1°, 15.1° ± 4.5° and 12.5° ± 6.6°, respectively, as shown in Fig. 3(a). Since loading P3HT at 50% led to the formation of grass-like, curved TIPS pentacene crystals as can be easily seen from Fig. 2(d), the misorientation angle method wasn’t applied to characterize the crystal random orientation at this ratio. Therefore, it can be inferred that the addition of the P3HT conjugated polymer in general reduced the crystal misorientation of TIPS pentacene film, and specially, P3HT at 2% of loading significantly decreased the misorientation angle to below 10°, implying that the crystal orientation and uniformity of the TIPS pentacene/P3HT blend film was greatly improved in contrast to the pristine TIPS pentacene crystals.

In addition to tuning the crystal orientation, the addition of the P3HT conjugated polymer additive also modulated the grain width of TIPS pentacene film. We define the grain width, or *W*_*G*_, as the crystal domain width along the short axis [1$\bar{2}$0] of a TIPS pentacene crystal, as shown in the inset of Fig. 3(b). While the pristine TIPS pentacene film exhibited a large grain width of 91 ± 20 µm as previously reported^17^, the grain width changed to 17.2 ± 6.92 µm, 8.35 ± 2.39 µm, 4.75 ± 0.93 µm, and 9.41 ± 1.87 µm, at the P3HT loading ratio of 2%, 10%, 25% and 50%, respectively, as shown in Fig. 3(b). Since crystalline defects and charge trap centers are located at the grain boundaries, reduced grain width indicates enhanced grain boundary densities, which can be unfavorable for the charge transport of TIPS pentacene crystals^36^. It can be seen that both misorientation angle and grain width of TIPS pentacene were dependent on the loading ratios of the P3HT polymer additive. Different extents of intermolecular interactions between TIPS pentacene and P3HT were anticipated from different loading ratios of P3HT. Although it is not possible to quantify such intermolecular interactions, we believe this is the main factor that contributed to the specific values of misorientation angle and grain width as observed in Fig. 3.

**Figure 4:**
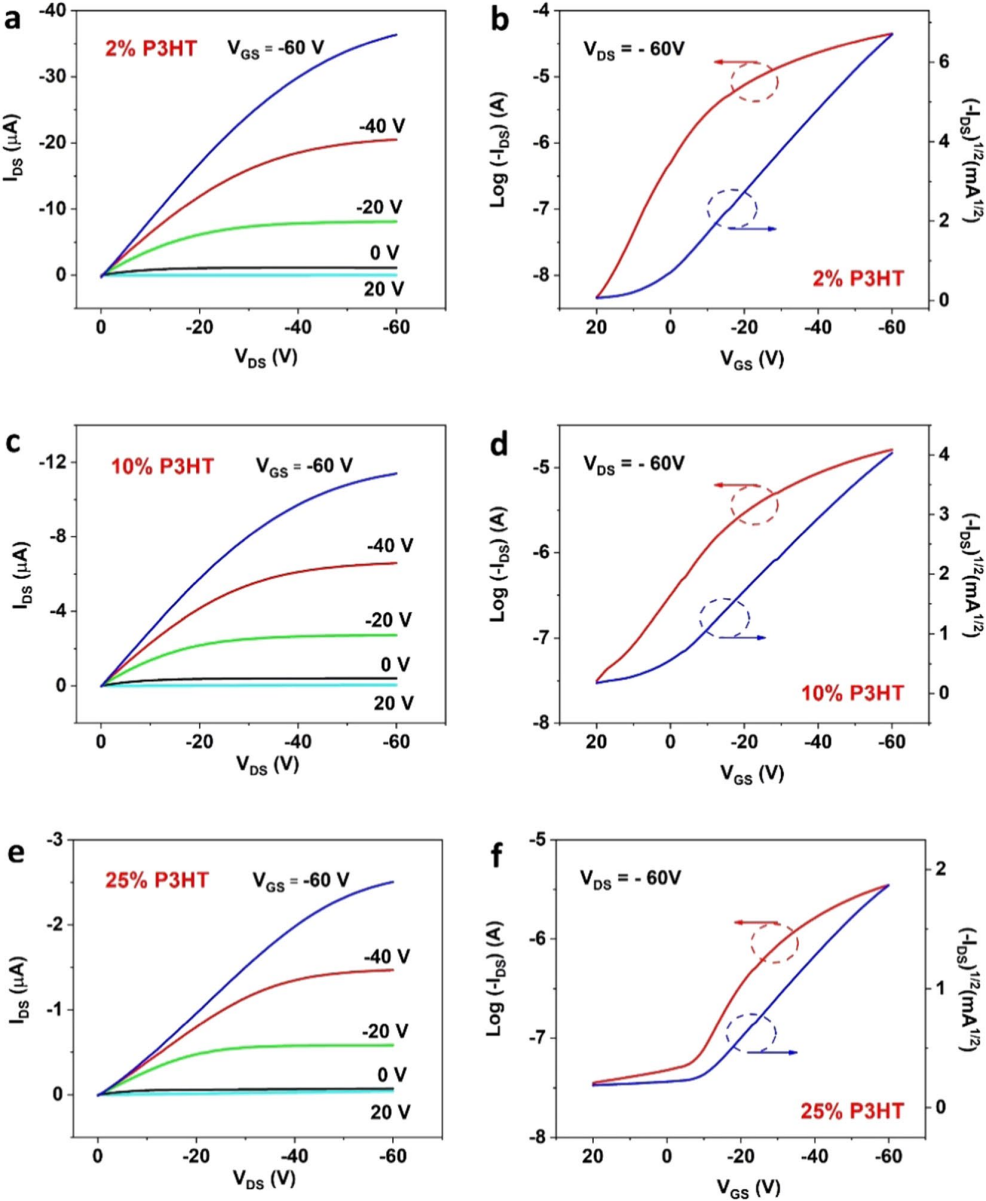
Representative (**a,c,e**) output and (**b,d,f**) transfer characteristics of bottom-gate, top-contact TIPS pentacene OTFTs with different loading ratios of P3HT, including 2%, 10% and 25%. Field-effect mobilities were extracted from the square root plot of the transfer characteristic in the saturation region at V_DS_ = −60 V.

Small-molecule TIPS pentacene/conjugated polymer P3HT hybrid OTFTs with a bottom-gate, top-contact configuration (Fig. 1(c)) were fabricated to characterize the mobility variation and performance consistency of the semiconductor due to the reduced crystal random orientation. Since TIPS pentacene film with 50% P3HT additive exhibited grass-like, curved microwires with elevated misorientation, the following electrical characterization of OTFTs was only based on the P3HT ratio of 2%, 10% and 25%. The typical OTFT output and transfer characteristics were represented in Fig. 4, as a function of different P3HT ratios. The field-effect mobilities of TIPS pentacene based OTFTs can be extracted from the square root plot of the transfer curve, based on Eq. (1):

1 $${{I}}_{{DS}}={\mu }{{C}}_{{i}}\frac{{W}}{2{L}}{({{V}}_{{GS}}-{{V}}_{{T}})}^{2}$$

where *μ* is the field-effect mobility, *C*_*i*_ is the SiO_2_ gate dielectrics capacitance (13.8 nF/cm^2^), *W* and *L* are the channel width and length, and *V*_*T*_ is the threshold voltage.

The extracted mobility from the transfer curves in Fig. 4, the current on/off ratio, threshold voltage, and channel width and length, were summarized in Table 1. Although a different channel width/length (W/L) ratio was used in this work from the W/L ratio in our previous work^17^, it is important to note that the channel dimension defined by the shadow mask, rather than the actual channel dimension covered by the crystals, was used to measure the mobilities. The different coverage in the channel region can contribute to the calculated mobilities. A higher areal coverage of the TIPS pentacene/P3HT blend film can result in improved mobilities.

**Table 1:** Summary of the extracted mobility (*µ*) from the transfer curves in Fig. 4, and the corresponding current on/off ratio, threshold voltage (*V*_*t*_) and channel width and length (*W/L*). The loading ratios of P3HT were 2%, 10% and 25%, respectively.

P3HT Ratio	Extracted *µ* (cm^2^/Vs)	On/Off Ratio	*V*_*t*_ (V)	*W/L* (µm)
2%	0.0896	9.546E + 03	−4.2	2000/100
10%	0.0268	5.177E + 02	−8.0	2000/100
25%	0.0041	9.885E + 01	2.5	2000/50

**Figure 5:**
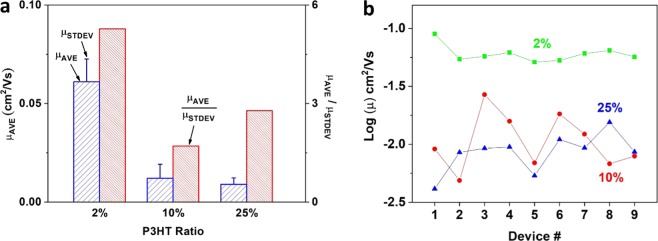
(**a**) Plot of mobility variations of OTFTs with different P3HT weight ratios. (**b**) Comparison of average mobility (*μ*_*AVE*_) with standard deviation (*μ*_*STDEV*_), as well as device performance consistency (represented by *μ*_*AVE*_/*μ*_*STDEV*_).

The average mobility (*μ*_*AVE*_) of TIPS pentacene OTFTs with different loading ratios of P3HT polymer additive was compared in Fig. 5(a). In contrast, the devices with 2%, 10% and 25% loading ratios of P3HT exhibited an average mobility of 0.061 ± 0.012 cm^2^/Vs, 0.012 ± 0.007 cm^2^/Vs, and 0.009 ± 0.003 cm^2^/Vs, respectively. This indicates that the addition of 2% P3HT doubled the average mobility of TIPS pentacene OTFTs. Specially, a hole mobility of up to 8.96 × 10^−2^ cm^2^/Vs was demonstrated from the TIPS pentacene OTFTs with 2% P3HT additive, which was considerably higher than the mobility from 2 × 10^−3^ cm^2^/Vs to 1.37 × 10^−2^ cm^2^/Vs of spin-coated TIPS pentacene/P3HT blends film as reported in previous studies^37–39^.

As compared to various insulation polymers, P3HT as a *p*-type conjugated polymer was reported to have hole mobilities ranging from 10^−5^ cm^2^/Vs to 10^−4^ cm^2^/Vs^40^, while pure TIPS pentacene has an average hole mobility of 0.03 ± 0.03 cm^2^/Vs^17^. Therefore, the electrical conductivity of P3HT has a negligible effect on enhancing the charge transport in the TIPS pentacene/P3HT blend system. The mobility change at different P3HT ratios was a combined outcome of both reduced crystal misorientation and reduced grain width. As observed from Fig. 3, the addition of P3HT as a polymer additive reduced the misorientation angle and grain width of TIPS pentacene crystals. The improved mobility at 2% P3HT can be mainly attributed to its very small misorientation angle of 8.3° ± 4.1° at the expense of reduced grain width. However, at 10% and 25% P3HT loading, larger misorientation angles and even smaller grain width were both obtained. In this case, the benefit from reduced crystal misorientation may not offset the charge trapping effect from increased grain boundaries, which contribute to the reduced mobilities at 10% and 25% ratios.

It is well known that the measured field-effect mobilities are dependent upon many factors, such as the application of external alignment techniques (i.e. air flow navigation and solution shearing), different channel size, and utilization of surfactant treatment. Without external alignment, pristine TIPS pentacene based OTFTs with a large channel dimension only exhibited an average hole mobility of lower than 0.05 cm^2^/Vs, as previously reported^41,42^. It is thus important to note that a very large channel size (channel width of 2000 μm, and channel length of 25~100 μm) was used in this study in order to demonstrate the enhanced long-range order of TIPS pentacene crystals. Also, since the TIPS pentacene/P3HT mixture tended to severely dewet on a surfactant-treated, hydrophobic surface, no surfactant treatment was performed on the hydrophilic silicon dioxide surface to passivate the hydroxyl groups. Besides, the loading of P3HT polymer additive reduced the average grain width of TIPS pentacene crystals and consequently attributed to more defects and charge trap centers at grain boundaries. Finally, some crystals bridged the source-to-drain electrodes with an angle, as can be seen from the optical images in Fig. 2. Although this resulted in lower measured mobilities, the charge transport can be enhanced if the source-to-drain direction is manipulated to be in parallel with the crystal orientation.

In addition to the modulation of charge carrier mobility, we also evaluated how the addition of P3HT polymer tuned the mobility variations and performance consistency of TIPS pentacene based OTFTs. Figure 5(b) provides a clear comparison of the mobility variations at different loading ratios of P3HT polymer additive. While the field-effect mobilities of TIPS pentacene OTFTs without P3HT additive ranged from 8.4×10^−2^ cm^2^/Vs to 9.8×10^−5^ cm^2^/Vs^17^, the addition of P3HT reduced the mobility variation to 5.1×10^−2^ cm^2^/Vs – 8.96×10^−2^ cm^2^/Vs, 4.88×10^−3^ cm^2^/Vs – 2.68×10^−2^ cm^2^/Vs, and 4.13×10^−3^ cm^2^/Vs – 1.55×10^−2^ cm^2^/Vs, at the loading ratio of 2%, 10% and 25%, respectively. It can be concluded that while pristine TIPS pentacene OTFTs exhibited mobilities that varied by three orders of magnitude, the devices with the P3HT additive exhibited mobility variation within one order of magnitude. Apparently, the addition of P3HT conjugated polymer has in general reduced the variation of mobilities of TIPS pentacene crystals.

To better characterize the effect of P3HT additive on the performance consistency of TIPS pentacene OTFTs, we continue to define the ratio of average mobility to standard deviation of mobility (*μ*_*AVE*_/*μ*_*STDEV*_) as an evaluation metric. Without the P3HT additive, the pristine TIPS pentacene OTFTs had a ratio factor of 1, implying anisotropic charge transport from randomly-oriented TIPS pentacene crystals. In contrast, the loading of P3HT polymer additive at 2%, 10% and 25% weight ratios increased the ratio factor to 5.27, 1.71, and 2.78, respectively, as shown in Fig. 5(a). In particular, it led to a 5-fold enhancement of the *μ*_*AVE*_/*μ*_*STDEV*_ ratio at 2% loading of P3HT additive, which is indicative of a significant improvement of performance consistency of TIPS pentacene based OTFTs.

**Figure 6:**
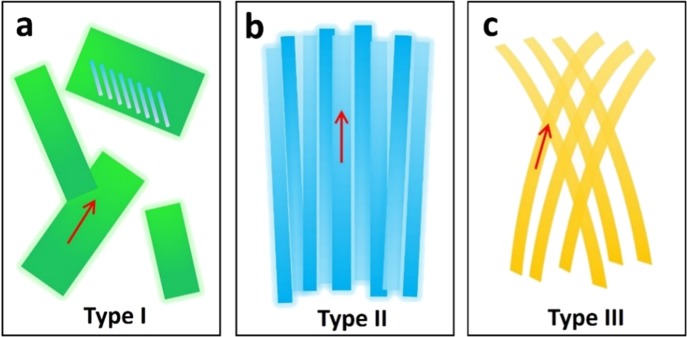
A schematic that shows each type of TIPS pentacene morphology. (**a**) Type I: TIPS pentacene crystals with random orientation and poor film coverage. (**b**) Type II: elongated TIPS pentacene crystals along a uniform direction with improved long-range order and areal coverage. (**c**) Type III: grass-like, curved TIPS pentacene microwires with interlinkage. The tilting blue rods in (**a**) indicate the orientation of TIPS pentacene backbones. The red arrows in (**a**–**c**) indicate the long axis orientation of TIPS pentacene crystals.

We use the schematic in Fig. 6 to illustrate the morphology change of TIPS pentacene thin film as a result of adding P3HT conjugated polymer additive. As Fig. 6(a) shows, the pristine TIPS pentacene crystals were severely misoriented with large gaps in between. The charge transport based on such misoriented TIPS pentacene crystals is anisotropic and results in significant device performance variations of OTFTs. We define the pristine TIPS pentacene film as a “Type I” morphology. When P3HT was added at a small amount (i.e. at a weight ratio of 2%), the conjugated polymer additive led to elongated needles with uniform crystal orientation and enhanced coverage, which we define as a “Type II” morphology in Fig. 6(b). When the loading ratio of P3HT increases to 50%, it led to the dominant formation of grass-like curved microwires with interlinkage, as shown in the “Type III” morphology in Fig. 6(c). On the other hand, when P3HT was added at an intermediate ratio (i.e. 25%), it caused a transitional morphology between “Type II” and “Type III”, which can be characterized by a mixture of both crystal needles and curved microwires. Each different type of TIPS pentacene thin film morphology exhibited distinctive crystal orientation and uniformity, grain width and areal coverage, which also attributed to different charge transport and device performance consistency of TIPS pentacene based OTFTs.

The successful tuning of TIPS pentacene thin film morphology from Type I to III can be accredited to both the intermolecular interactions between TIPS pentacene and P3HT. When P3HT was mixed with TIPS pentacene, there were two dominant kinds of intermolecular interactions, including hydrophobic interaction from the side chains of P3HT and the triisopropylsilylethynyl side groups of TIPS pentacene, as well as π−π interaction from the polymer additive and the semiconductor backbones. Such intermolecular interactions largely dictated the crystallization and resultant thin film morphology of TIPS pentacene. In the early stage of crystallization, the formation of aggregated cluster and kinetics of heterogeneous nucleation play a crucial part in polymorphism, the intermolecular interactions can largely impact the final polymorphism and morphology^43–45^. As a result, simply by varying the loading ratio of the conjugated polymer additive, the extent of intermolecular interactions between TIPS pentacene and P3HT can be modulated, which contributes to different film morphology and charge transport of TIPS pentacene OTFTs.

Finally, it is important to note that these types of TIPS pentacene film morphology as demonstrated in this work are not obtainable by solely using external field alignment techniques or adding non-conjugated polymer additives. External alignment methods, such as air flow navigation and solution shearing, in general only improve the crystal orientation of the randomly-oriented TIPS pentacene needles, whereas various non-conjugated polymer additives either disperse the crystal needles by serving as a matrix or induce a vertical phase separation mainly dependent on the entropic or hydrophobic forces. Therefore, conjugated polymer assisted organic semiconductor crystallization, facilitated by the intermolecular interaction between TIPS pentacene and P3HT conjugated polymer additive, offers an exclusively novel pathway to fine-tune crystallization, thin film morphology and charge transport of TIPS pentacene OTFTs. Since the characterization of such intermolecular interactions is beyond the scope of this work, a further study is needed to provide an in-depth understanding.

## Conclusions

To conclude, we have reported an effective approach to control the growth and morphology of a solution processed, small-molecule organic semiconductor TIPS pentacene by blending P3HT as a conjugated polymer additive. By varying the weight ratio of P3HT, three different types of TIPS pentacene film morphology were obtained, including the randomly-oriented crystal ribbons, elongated needles with enhanced long-range order, and grass-like curved microwires with interlinkage. Correspondingly, each type of morphology correlated to different semiconductor crystallization and charge transport of TIPS pentacene crystals. Bottom-gate, top-contact OTFTs based on TIPS pentacene/P3HT hybrid film exhibited a 2-fold and 5-fold enhancement of average mobility and performance consistency, as compared to the pristine TIPS pentacene OTFTs. The successful modulation of semiconductor morphology and mobility with the addition of conjugated polymer additive can be accredited to the intermolecular interactions between the semiconductor and polymer, including the hydrophobic interaction from their side chains and π−π interactions from their backbone. This work offers a novel pathway to improve the charge transport of other solution-processed, small-molecule organic semiconductors in general.
